# A Sanitary Shibboleth.—Small-Pox Infection

**Published:** 1896-04-18

**Authors:** 


### A Sanitary Shibboleth.—Small-pox Infection.

To the medical mind there are few things more objectionable than attempts to limit freedom of thought and action by the imposition of creeds and dogmas. We need not wonder, then, that medical men are ready to protest when they see reason to believe that acceptance of certain official sanitary shibboleths is becoming essential in those who wish for official recognition and advancement.

The particular dogma which is at present in high favour in official quarters is that of the aerial diffusion of small-pox infection, or infection through the air at a distance—that distance running up to at least a mile—and it is believed by many that those who refuse to bow the knee to this Whitehall Baal will receive short shrift and small encouragement from the ruling powers at the Local Government Board.

Our attention is drawn to this matter by the Milroy Lectures, delivered recently before the Royal College of Physicians, in which Dr. Seaton throws some doubt upon the correctness of the official view.

Now nothing would be further from our wish than to say that such a mode of distribution is not possible, or to throw any slight on the painstaking carefulness with which Dr. Power conducted those historic investigations on which the hypothesis of aerial convection was first founded; nevertheless, in view of the attention which has been drawn to the question by many recent events, we would point out the very large consequences which must follow from that unreserved acceptance of it which seems at present to be expected from those who adopt public health as their department of practice.

It was proved that the admission of acute cases of small-pox to the Fulham Hospital was followed by the occurrence of cases in the surrounding districts, as has also been proved in regard to other outbreaks, and that the proportion of cases in the several zones around the hospital diminished as the distance from it increased, and we may admit that, if it were proved that the infection of small-pox could be carried by the air for the distance of a mile, these might be taken as very good examples of such aerial convection, yet it is clear that just where proof is wanted there the proof stops short. It is officially maintained that because no other explanation has been offered of this zonular arrangement of the cases, which was not equally, if not more, difficult of acceptance, therefore the infection must have been carried by the air. But it is obvious enough that the very essence of such a form of proof lies in the proved absence of all other possible alternatives—the very thing which in this case was taken for granted. We may then be content to agree with Mr. Justice Kekewich, who in a recent decision refused to admit that the theory of aerial convection was sufficiently established to enable the court to say that such a mode of infection was a danger which must be provided against. We emphasise the fact that the whole theory is founded on the assumption that no other explanation can be found, and we drew attention to the very large consequences which must follow if this theory should be accepted and logically acted upon by the sanitary authorities of the country. All who accept the theory must be prepared to act as if for a mile, and possibly for some unknown further distance, infection may be spread through the air from even a well-managed small-pox hospital. This means that it must soon become impossible for sanitary authorities to obtain sites for such institutions, unless they can discover blocks of uninhabited land a couple of miles across, which is obviously impossible, or obtain such control over these wide areas as to prevent their being visited by susceptible people, a thing almost equally out of the question.

It is an interesting matter, worthy of the most careful consideration, that, while sanitary authorities are crying out about the difficulties they experience in obtaining sites, and are declaiming against the selfishness of private owners in preventing the erection of small-pox hospitals in their neighbourhood, it is on the evidence and the theories of the highest official sanitary authorities of the country that these private owners rely for the proof that such hospitals must, by virtue of the aerial diffusion of infection from them, be nuisances injurious to health.

Much more might be said, but we have said enough to show that this theory or hypothesis of aerial convection of small-pox, however true it may turn out to be, is far from being so self-evident a verity as to make it a fitting test of sanitary orthodoxy, and we would suggest that the Local Government Board might do better by publishing all the evidence it possesses on the other side than by insisting on the impugnablity of the position it has taken up. In the meantime our thanks are due to Dr. Seaton for drawing attention to the tendency of official documents to refer over and over again to the same set of facts and thus to give rise to an exaggerated impression of their importance.

